# Single-Cell Profiling Reveals the Origin of Phenotypic Variability in Adipogenesis

**DOI:** 10.1371/journal.pone.0005189

**Published:** 2009-04-09

**Authors:** Thuc T. Le, Ji-Xin Cheng

**Affiliations:** 1 Weldon School of Biomedical Engineering, Purdue University, West Lafayette, Indiana, United States of America; 2 Department of Chemistry, Purdue University, West Lafayette, Indiana, United States of America; Department of Medicine, University of Hong Kong, China

## Abstract

Phenotypic heterogeneity in a clonal cell population is a well-observed but poorly understood phenomenon. Here, a single-cell approach is employed to investigate non-mutative causes of phenotypic heterogeneity during the differentiation of 3T3-L1 cells into fat cells. Using coherent anti-Stokes Raman scattering microscopy and flow cytometry, adipogenic gene expression, insulin signaling, and glucose import are visualized simultaneously with lipid droplet accumulation in single cells. Expression of adipogenic genes PPARγ, C/EBPα, aP2, LP2 suggests a commitment to fat cell differentiation in all cells. However, the lack of lipid droplet in many differentiating cells suggests adipogenic gene expression is insufficient for lipid droplet formation. Instead, cell-to-cell variability in lipid droplet formation is dependent on the cascade responses of an insulin signaling pathway which includes insulin sensitivity, kinase activity, glucose import, expression of an insulin degradation enzyme, and insulin degradation rate. Increased and prolonged insulin stimulation promotes lipid droplet accumulation in all differentiating cells. Single-cell profiling reveals the kinetics of an insulin signaling cascade as the origin of phenotypic variability in drug-inducible adipogenesis.

## Introduction

Targeting adipose tissues to reduce adipose mass has been proposed as a viable approach to obesity treatment [1]–[3]. However, variability in adipogenesis activity among preadipocytes presents significant challenges to drug intervention [4]. Cells with distinct molecular profile display distinct phenotypic behavior and drug response [5], [6]. To improve the efficacy of drug treatment, the source of cell-to-cell variability should be identified and targeted [7]. Unlike genetic mutation which can be identified with sequencing, non-genetic cause of cell-to-cell variability cannot be readily investigated. Standard population measurement techniques describe average behavior and are insufficient to investigate variability among cells [8]. To describe the molecular cause of phenotypic variability, cellular events and phenotypic expression must be measured simultaneously at the level of single cells [9], [10]. Such requirement demands multiple signals to be analyzed at the same time within a single cell.

There exist a number of powerful techniques for single-cell analysis including microscope-based cytometry [11] and flow cytometry [12]. Traditionally, these techniques analyze the expression of fluorescent molecules. However, technical challenges associated with exogenous fluorescent labeling combined with limited endogenous fluorescent molecules place a constraint on the number of assayable molecules within a single cell. More recently, coherent anti-Stokes Raman scattering (CARS) microscopy with its label-free imaging capability has expanded the number of biomolecular structures which can be optically examined [13], [14]. Being highly sensitive to CH_2_ vibration of lipid-rich molecules, CARS microscopy has been applied to image lipid domains, axonal myelin sheaths, lipid-rich foam cells, and lipid-rich metastatic cancer cells [15]–[18]. CARS imaging has also been applied to study adipogenesis and lipid droplets lipolysis in 3T3-L1 cells [1], [19], lipid droplets trafficking in adrenal cortical Y-1 cells [20], and lipid storage in *Caenorhabditis elegans* [21]. Microfluidic CARS flow cytometry further enables label-free high throughput analysis of lipid droplets of mature adipocytes [22]. A unique advantage of a CARS microscope is its capability for multimodal nonlinear optical imaging on a single platform including CARS and two-photon excitation fluorescence (TPEF) [23]. Combining fluorescent imaging with vibrational imaging, a typical CARS microscope is capable of monitoring multiple signals simultaneously. This capability renders CARS microscopy an ideal tool to study the molecular cause of cell-to-cell phenotypic variability.

**Figure 1:**
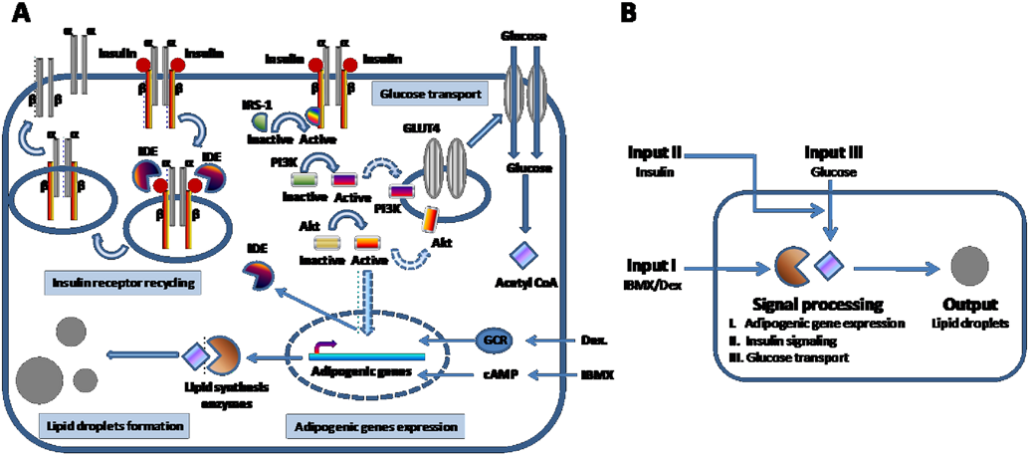
A simplified view of a drug-inducible adipogenesis system. (A) The effects of adipogenic stimulants on adipogenesis. IBMX and dexamethasone induce expression of lipid synthesis enzymes. Insulin activates kinase signaling pathway leading to glucose import. A product of glycolysis, acetyl CoA, serves as a substrate for lipid synthesis enzymes. Akt kinase activates expression of insulin degradation enzyme (IDE) which allows recycling of surface insulin receptor. (B) 3T3-L1 cells as single computing units. Inputs are defined as IBMX/dexamethasone, insulin, and glucose. Signal processing is defined as cellular activities induced by input signals which include adipogenic gene expression, insulin signaling, and insulin-stimulated glucose import. Output is defined as intracellular lipid droplets formation.

To study adipogenesis, a well-established cell culture model using 3T3-L1 cell line has been employed since 1974 [24]. Adipogenesis in 3T3-L1 cells are initiated with a mixture of adipogenic stimulants isobutylmethylxanthine (IBMX) and dexamethasone which turn on transcription factor PPARγ to direct the transcription of lipid synthesis genes. Then, insulin is added to facilitate glucose uptake and promote adipocyte differentiation (**Fig. 1A**) [25]. Because adipogenesis in 3T3-L1 cells has been well-characterized, it is commonly used to study the effects of drugs on fat cell differentiation [2]. However, heterogeneity in the rates of lipid droplet formation among cells prevents direct evaluation of the drug efficacy [4]. Heterogeneity in adipogenesis was first described by Green and Kehinde in 1974 in their original 3T3-L1 cell cultures and became a well-documented phenomenon since then (**Fig. S1**) [24]. Using 3T3-L1 cell cultures, the molecular controls of adipogenesis pathway have been well-elucidated [3]. Nonetheless, the molecular cause of cell-to-cell variability during adipogenesis remains a mystery.

Taking advantage of the multimodality of a typical CARS microscope, we investigate the cause of cell-to-cell variability during adipogenesis by imaging multiple cellular events simultaneously within single 3T3-L1 cells. Imaging data are further complemented by high-throughput quantitative single-cell analysis by flow cytometry. We treat each 3T3-L1 cell as a single computing unit by defining input signals as adipogenic stimulants IBMX, dexamethasone, and insulin. Signal processing is defined as expression of adipogenic genes, insulin signaling, or glucose import. And output signal is defined as the rate of lipid droplet formation as determined by the quantity and size of cytoplasmic lipid droplets (**Fig. 1B**). By keeping input signals constant, we simultaneously measure signal processing and output signals within single cells. Correlating signal processing to variations in output signal would allow identification of cellular events which are responsible for phenotypic variability among 3T3-L1 cells in drug-inducible adipogenesis.

## Results

### Monitoring adipogenesis with CARS imaging, real-time PCR, and flow cytometry

**Figure 2:**
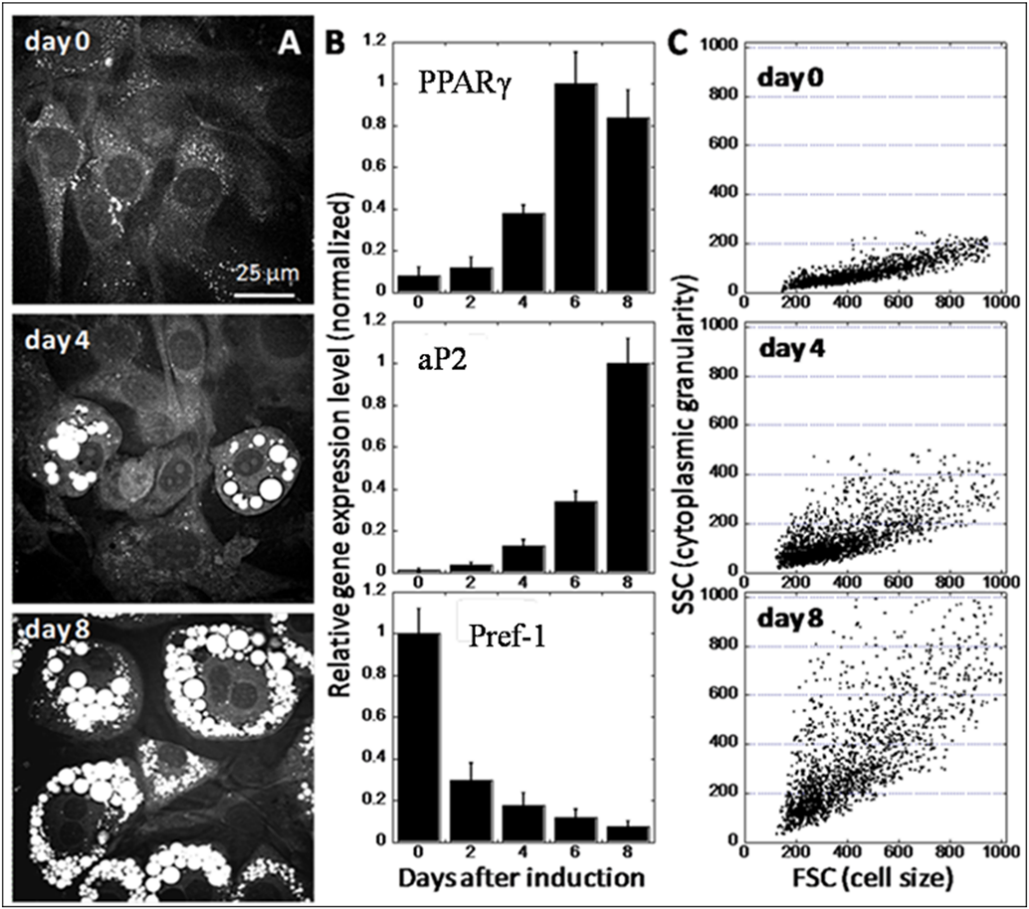
Monitoring adipogenesis with CARS microscopy, real-time PCR, and flow cytometry. (A) CARS visualization of intracellular lipid droplets accumulation as a function of time after adipogenesis induction. For CARS imaging, increased lipid droplet accumulation is due to increased of lipid amount in each cell. (B) Real-time PCR gene expression profiling of adipogenic gene markers as a function of time after adipogenesis induction. Adipogenic gene expression levels were corrected with expression level of a house-keeping gene acidic ribosomal phosphoprotein and normalized to 1 with the highest expression level. Error bars represent distribution across four repeated experiments. (C) Flow cytometry analysis of side scattering signal (SSC) which indicates cytoplasmic granularity caused by lipid droplets accumulation and forward scattering signal which indicates cell size.

Adipogenesis in 3T3-L1 cells can be measured using a number of techniques including CARS microscopy [1], [19], adipogenic gene expression profiling [3], and flow cytometry [4]. Using CARS microscopy, adipogenesis is monitored by the quantity of lipid droplets accumulation within the cell membrane (**Fig. 2A**). As CARS signal arises from CH_2_ vibration, lipid-rich structures such as cell membrane or lipid droplets yield positive contrast, whereas lipid-poor structures such as cell nuclei yield negative contrast. By integrating all positive contrasts within the cell membrane, the total lipid accumulation within a single cell can be inferred. Using CARS imaging, advanced-stage cells and early-stage differentiating cells can be defined as cells having high and low intracellular lipid accumulation, respectively. The degree of fat cell differentiation can also be measured by the expression level of lipid synthesis proteins or their controlling transcription factors (**Fig. 2B**) [3]. Among such proteins, increased expression of transcription factor PPARγ and its downstream target aP_2_ are required for adipogenesis [3]. Conversely, reduced expression of a membrane-bound surface protein, Pref-1, during adipogenesis is also observed (**Fig. 2B**) [26]. Alternatively, flow cytometry provides a high through-put means to assay lipid droplet formation (**Fig. 2C**). By measuring side-scatter signals emanating from passing cells, the degree of cytoplasmic granularity or size of lipid droplets of single cells can be obtained. Indeed, flow cytometry has been successfully used to sort 3T3-L1 cells based on the size of their cytoplasmic lipid droplets (**Fig. S2**) [4].

### Increased adipogenic genes expression does not necessitate lipid droplet formation

**Figure 3:**
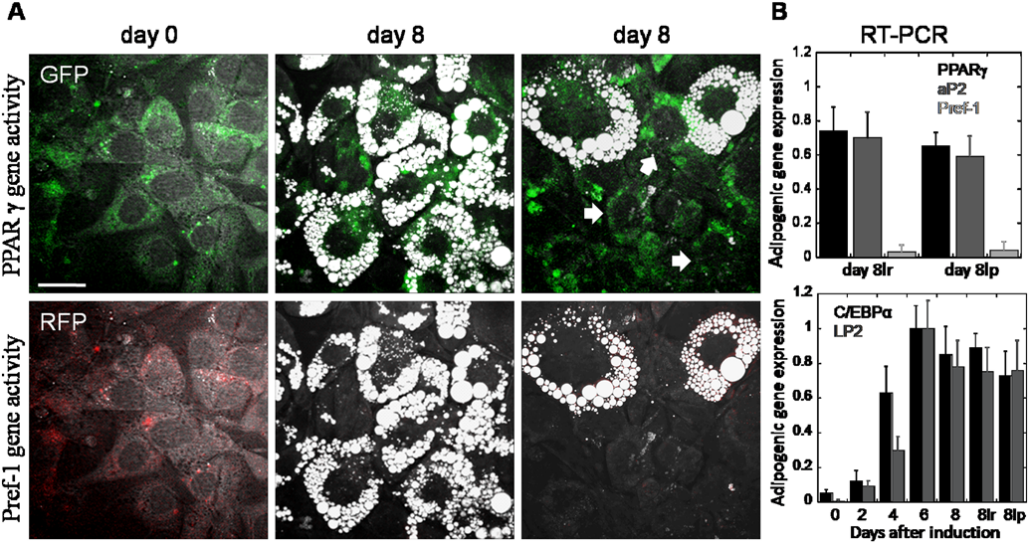
Increased adipogenic gene expression in both lipid-rich and lipid-poor cells. (A) Real-time visualization of PPARγ (upper panels, green) and Pref-1 (lower panels, red) gene activity using TPEF imaging of dsGFP (green) and dsRFP (red) signals at day 0 and day 8 after adipogenesis induction. Intracellular lipid droplets are visualized with CARS (grey) imaging. Arrows point to representative lipid-poor cells. Scale bar: 25 µm. (B) Real-time PCR gene expressions profiling of PPARγ, aP2, and Pref-1 gene activity (upper panel), and C/EBPα and LP2 gene activity (lower panel). PPARγ and C/BEPα are transcription factors whose expressions are critical for early-stage differentiation of 3T3-L1 cells. aP_2_ and LP2 are lipid-binding proteins whose expression are controlled by PPARγ. aP2 and LP2 expressions mark late-stage differentiation of 3T3-L1 cells. Pref-1 is a preadipocyte marker protein whose down-regulation is required for adipogenesis. 8lr and 8lp represent sorted lipid-rich and lipid-poor populations at day 8 after adipogenesis induction, respectively. Error bars represent distribution across 4 repeated experiments.

To determine whether IBMX and dexamethasone input signals processing is the source of variability in adipogenesis, we simultaneously examined adipogenic genes expression and lipid droplet formation within single 3T3-L1 cells. To monitor real-time gene expression in single cells, we stably transfected 3T3-L1 cells with a dual-reporter plasmid where the expression of destabilized variants of green fluorescent protein (dsGFP) and red fluorescent protein (dsRFP) are controlled by a PPARγ and a Pref-1 promoter, respectively (**Fig. 3A**) [27], [28]. Both dsGFP and dsRFP were tagged with ubiquitin destruction signals such that their half-lives were shortened to approximately 2 hours [29]. The rapid turnover rate of fluorescent proteins mimicked that of mRNA transcripts and allowed dynamic studies of PPARγ and Pref-1 promoter activity. PPAR*γ* is a nuclear factor required for gene transcription of many lipid synthesis enzymes. Increased expression of PPAR*γ* gene has previously been shown to be *sine qua non* for adipogenesis [3]. Conversely, Pref-1 is a surface membrane bound protein whose down-regulation is required for adipogenesis [26]. Simultaneous measurement of PPARγ and Pref-1 promoter activity serves as a self-calibrated indicator of adipogenic genes expression.

Using TPEF to image PPARγ and Pref-1 promoter activity and CARS to image intracellular lipid droplets, we observed increased adipogenic gene activity did not always correlate with lipid droplet accumulation. Following IBMX and dexamethasone stimulation, a significant increase in PPARγ gene activity and a strong suppression of Pref-1gene activity were observed in lipid-rich 3T3-L1 cells (**Fig. 3A**). However, increased PPARγ gene activity was also observed in lipid-poor 3T3-L1 cells (**Fig. 3A**). Increased PPARγ gene activity was confirmed with real-time PCR profiling of PPARγ gene transcripts of lipid-rich and lipid-poor 3T3-L1 cell populations sorted with flow cytometry (**Fig. 3B**). Expression of a PPARγ downstream target gene aP2 further indicates an increase in PPARγ protein function in both lipid-rich and lipid-poor cells (**Fig. 3B**). More convincingly, early-stage marker genes including PPARγ and C/EBPα and late-stage marker genes including aP2 and LP2 were fully expressed in all cells which suggest commitments to fat cell differentiation in both lipid-rich and lipid-poor cells (**Fig. 3B**). We further examined the relationship between PPAR*γ* protein activity and lipid droplet accumulation by adding to the differentiating cell cultures a PPAR*γ* agonist, rosiglitazone [30]. We observed rosiglitazone addition increases the amount of lipid droplets in lipid-rich cells (**Fig. S3**). However, rosiglitazone addition had no impact on the percentage of 3T3-L1 cells with cytoplasmic lipid droplets (**Fig. S3**). This observation suggests that neither increased adipogenic gene expression nor increased PPARγ protein activity necessitate lipid droplet accumulation.

### Increased insulin degradation rates in lipid-rich cells

**Figure 4:**
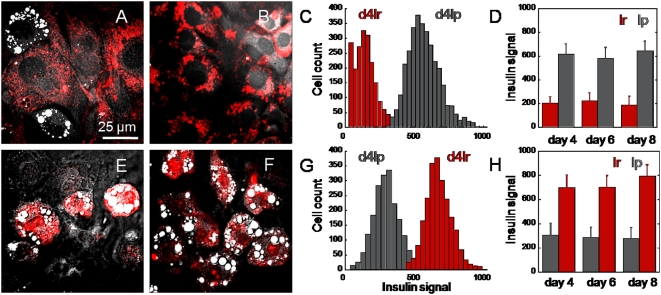
Insensitivity to new insulin stimulation in lipid-poor cells. (A) Absence and (B) presence of insulin-Cy3 signal in lipid-rich and lipid-poor cells, respectively. Cells were treated with insulin-Cy3 on day 2 and images taken on day 4. (C) Flow cytometry analysis of insulin-Cy3 signal in lipid-rich (red) and lipid-poor cells (grey) of cell samples presented in (A) and (B). 4lr and 4lp represent sorted lipid-rich and lipid-poor populations at day 4 after adipogenesis induction, respectively. (D) Quantitative analysis of insulin-Cy3 signal in lipid-rich (lr) and lipid-poor (lp) sorted cell populations at different days after adipogenesis induction. Insulin-Cy3 was added every 2 days to cell cultures until day 4 and day 6 for analysis on day 6 and day 8, respectively. Error bars represent distribution across 4 repeated experiments. Two thousand cells were evaluated with flow cytometry for each experiment. (E) Lipid-poor cells exhibit insulin resistance, whereas (F) lipid-rich cells exhibit insulin sensitivity to additional insulin stimulation. Cells were treated with unlabeled insulin on day 2, then with insulin-Cy3 on day 4 for 5 minutes before imaging. (G) Flow cytometry analysis of insulin-Cy3 signal in lipid-rich (red) and lipid-poor cells (grey) of cell samples presented in (E) and (F). (H) Quantitative analysis of insulin-Cy3 signal in lipid-rich (lr) and lipid-poor (lp) sorted cell populations at different days after adipogenesis induction. Cells were treated with unlabeled insulin every 2 days until the day of analysis, when insulin-Cy3 was added for 5 minutes before imaging. Error bars represent distribution across 4 repeated experiments. Two thousand cells were evaluated with flow cytometry for each experiment.

Next, we examined the correlation between insulin signal processing and variability in lipid droplet accumulation of 3T3-L1 cells. We used fluorescently-labeled insulin which had been widely used with no reported perturbation to its intended function [31]. At two days after insulin stimulation, we observed lipid accumulation in many 3T3-L1 cells (**Fig. 4A**). Whereas no insulin fluorescent signal was detected in lipid-rich cells, significant insulin fluorescent signals were observed in lipid-poor cells (**Fig. 4A–D**). This observation was striking given the effect of insulin-induced lipid droplet accumulation was evident in lipid-rich cells. Because insulin was quickly degraded after cellular uptake [32], we hypothesized that lipid-rich cells degrade insulin at a faster rate than lipid-poor cells. To test this hypothesis, we first stimulated cells with unlabeled insulin for two days, then added labeled insulin. After 5 minutes, all lipid-rich cells quickly exhibited labeled insulin (**Fig. 4E–H**); whereas, lipid-poor cells remained resistant to the new source of labeled insulin (**Fig. 4E**). The inability of lipid-poor cells to uptake new source of labeled insulin can be attributed to competitive binding between unlabeled and labeled insulin (**Fig. S4**). The continued presence of previous source of unlabeled insulin in lipid-poor cells prevented further uptake of new source of labeled insulin (**Fig. 4B, E**).

**Figure 5 pone-0005189-g005:**
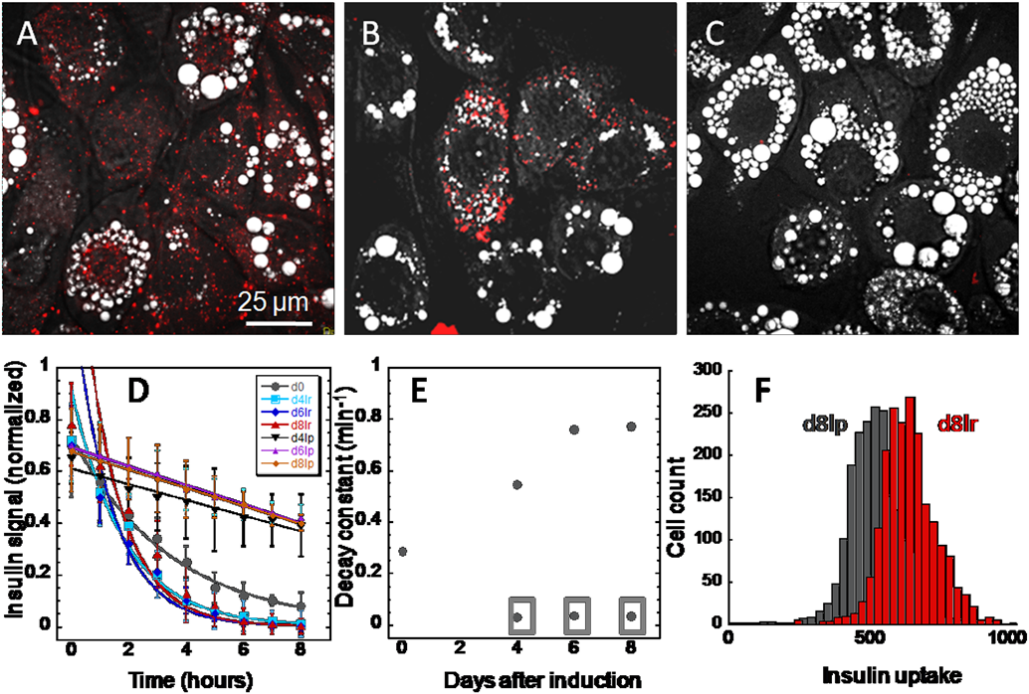
Increased insulin degradation rate in lipid-rich cells. (A) Insulin uptake in both lipid-rich and lipid-poor cells at 5 minutes after insulin-Cy3 addition. (B) Insulin signal degrades faster in cells with more lipid droplet accumulation at 4 hours after insulin-Cy3 addition. (C) Insulin signal is undetectable in lipid-rich cells at 6 hours after insulin-Cy3 addition. (A–C) Cells on day 6, or 2 days after removal of unlabeled insulin, are used. (D) Quantitative analysis of insulin signal degradation in representative cells at various days after induction. Error bars represent distribution of insulin signal across 20 representative cells analyzed. (E) Decay constant extracted from (D) is plotted as a function of time after induction. (F) Flow cytometry analysis shows increase in insulin uptake of lipid-rich cells (red) as compared to lipid-poor cells (grey) at day 8 after induction. Red: TPEF insulin-Cy3 signal. Grey: CARS lipid signal.

Tracking of labeled insulin in 3T3-L1 cells over time revealed the relationship between insulin degradation and lipid droplet accumulation. To compare insulin degradation rates between lipid-rich and lipid-poor cells, we used a cell culture at 6 days after adipogenesis initiation. At this time, insulin source from culture media had been removed for 2 days; hence, no insulin should remain in lipid-rich or lipid-poor cells. We added labeled insulin and monitor insulin signal over time. At 5 minutes after addition, we found insulin signals in both lipid-rich and lipid-poor cells (**Fig. 5A**). In lipid-rich cells, the rate of insulin signal degradation was more rapid in cells with high lipid accumulation than in cells with low lipid accumulation (**Fig. 5B**). At 6 hours after addition, insulin signal degraded to nearly undetectable level in lipid-rich cells (**Fig. 5C**). This observation clearly supports our hypothesis of fast insulin degradation rate in lipid-rich cells (**Fig. 4A**).

We further quantitatively analyzed the degradation rates of insulin in cells at various days after adipogenesis induction. First, we defined a number of criteria for this quantitative analysis of insulin degradation rates. Cells stimulated with IBMX/Dex stimulants from day 0 to day 2 were treated with 10 µg/ml labeled insulin from day 2 to day 4. To analyze insulin degradation rate, labeled insulin was removed on day 4. Then, cells were exposed to 10 µg/ml of new labeled insulin source for 5 minutes on specified days. Next, cells were washed thoroughly to remove exogenous labeled insulin and monitored over time for the presence of internalized labeled insulin. We defined representative lipid-rich cells in cell cultures at 4, 6, and 8 days after adipogenesis induction as cells having lipid droplets of approximately 2 µm, 4 µm, and 6 µm in diameters, respectively. Lipid-poor cells were defined as cells devoid of observable lipid droplets in all time points. These criteria allowed comparison of insulin degradation rates of cells at various days after adipogenesis induction with minimal interference from cell-to-cell variability. Second, fluorescent insulin signals were collected from 20 representative cells at 1-hour time intervals for 8 hours. Average insulin fluorescent intensity and the distribution across 20 cells were plotted as a function of time after labeled insulin addition (**Fig. 5D**). We avoided measurements within the same cells to minimize photo-bleaching of insulin signal caused by repeated exposure to laser beams.

We found strong correlations between insulin degradation rates and days after adipogenesis induction. In lipid-rich cells, insulin degradation could be fitted with a first-order decay function (**Fig. 5D**); whereas, in lipid-poor cells, insulin degradation could be described with a linear decay function (**Fig. 5D**). Decay constant values for lipid-rich cells increased as a function of days after adipogenesis induction (**Fig. 5E**). On the contrary, decay constant values for lipid-poor cells decreased as a function of days after adipogenesis induction (**Fig. 5E**). With regards to the ability to uptake insulin, we observed a slightly higher average insulin uptake signal in lipid-rich cells as compared to lipid-poor cells using flow cytometry analysis (**Fig. 5F** and **Fig. S5A**). This data is consistent with previous report of increased surface insulin receptors in differentiated 3T3-L1 cells [33]. Taken together, these observations further support our hypothesis of faster insulin degradation rates in lipid-rich cells as compared to lipid-poor cells.

### Increased kinase activity and glucose import in lipid-rich cells

**Figure 6 pone-0005189-g006:**
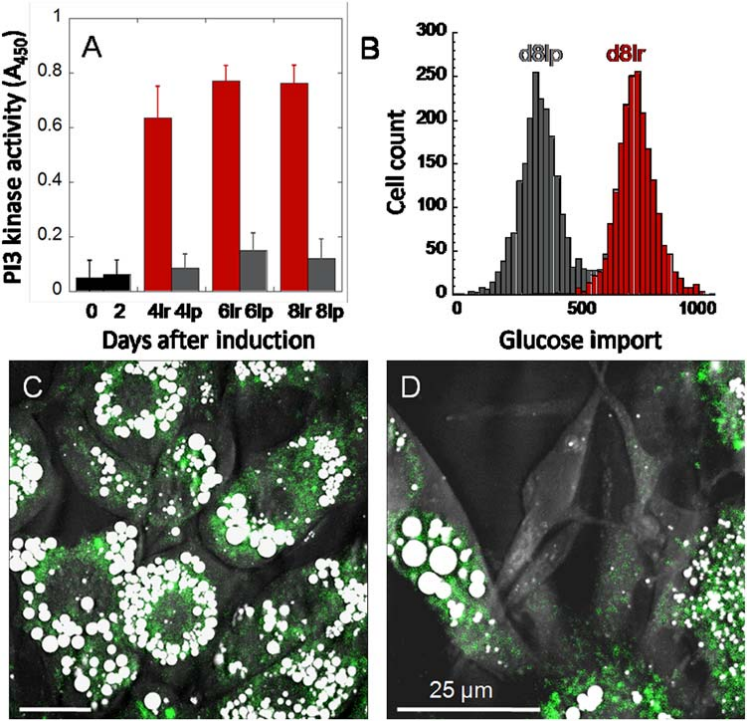
Increased kinase activity and glucose import in lipid-rich cells. (A) PI3K kinase activity of undifferentiated cells is significantly lower than differentiated cells. 8lr and 8lp represent sorted lipid-rich and lipid-poor cell populations at day 8 after adipogenesis induction, respectively. Error bars represent distribution across 4 repeated experiments. (B) Flow cytometry analysis of the import of a fluorescent glucose analog in a population of cells at day 8. Lipid-rich cells (red) exhibit approximately 2-fold higher in fluorescent glucose intensity than lipid-poor cells (grey). Imaging reveals strong glucose import in lipid-rich cells (C) as compared to lipid-poor cells (D). (C, D) Cells at day 6 are used for imaging. Scale bars: 25 µm.

Next, we examined the activity of downstream targets of an insulin signaling cascade. To evaluate insulin-stimulated kinase signaling activity, we measured the activation of phosphoinositide 3-kinase (PI3K) which is an immediately downstream target of insulin receptor substrate 1 (IRS-1) [25]. Using an ELISA-based assay to detect activation of receptor tyrosine kinase, we found approximately 20-fold increase of kinase activity in the lipid-rich cell population after insulin addition (**Fig. 6A**). However, a mere two-fold increase in kinase activity was observed in the lipid-poor cell population (**Fig. 6A**). This observation shows that lipid-poor cells exhibit a 10-fold reduction in kinase activity as compared to lipid-rich cells.

We further examined the activity of insulin-stimulated glucose import in 3T3-L1 cells. Using a fluorescent glucose analog [34], we analyzed imported glucose signal in lipid-rich and lipid-poor cells at day 8 using flow cytometry. On average, lipid-rich cells exhibited two-fold increase in fluorescent glucose analog uptake as compared to lipid-poor cells (**Fig. 6B** and **Fig. S5B**). Flow cytometry data was further supported by imaging data where strong and weak cytoplasmic fluorescent glucose analogs were observed in lipid-rich and lipid-poor cells, respectively (**Fig. 6C, D**). This observation suggests that increased kinase activity positively affects glucose import in lipid-rich cells. Increased glucose import would further increase the concentration of a glycolysis product, acetyl-CoA, which serves as a substrate for *de novo* lipid synthesis [**35**]. It is conceivable that the combined effects of increased insulin sensitivity, increased kinase activity, and increased glucose import would exponentially increase the rates of lipid droplet accumulation in lipid-rich cells.

### Increased expression of insulin degradation enzyme in lipid-rich cells

**Figure 7:**
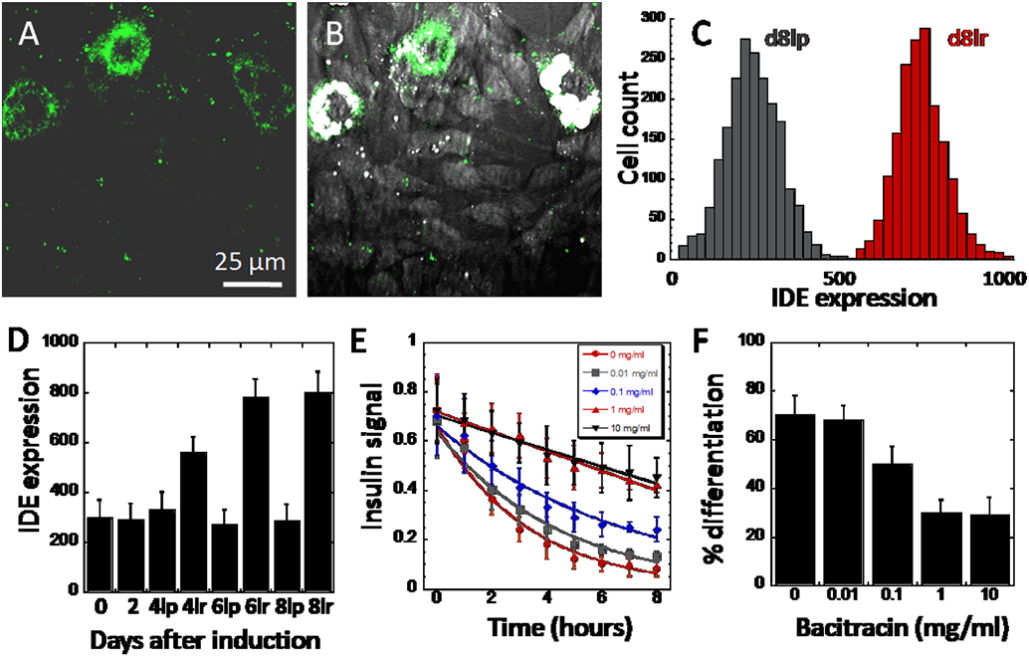
Increased expression of insulin degradation enzyme in lipid-rich cells. (A) TPEF imaging of fluorescent immuno-labeled insulin degradation enzyme in cells at day 8. (B) Overlay of TPEF imaging of IDE and CARS imaging of lipids. (C) Flow cytometry analysis of IDE expression level of lipid-rich cells (red) and lipid-poor cells (grey) at day 8. (D) Quantitative flow cytometry analysis of IDE expression level as a function of days after induction. lr and lp represent lipid-rich and lipid-poor cells at given days after induction. Error bars represent distribution across 4 repeated experiments. (E) Insulin signal decay as a function of bacitracin concentration. Undifferentiated cells at day 0 are used. (F) Percentage of cell differentiation as a function of bacitracin concentration. Error bars represent distribution across 4 repeated experiments.

From existing literatures, insulin degradation is controlled in part by insulin degradation enzyme (IDE) [32]. To investigate the role of IDE on the kinetics of insulin degradation of differentiating 3T3-L1 cells, we analyzed the expression of IDE using immuno-fluorescent labeling. Both imaging and flow cytometry analyses revealed a significantly higher level of IDE expression in lipid-rich as compared to lipid-poor cells (**Fig. 7A–D**). Increased IDE expression in lipid-rich cells was observed after insulin addition on day 2, which suggests a possible insulin-stimulated expression. Indeed, current literatures support the dependence of IDE expression on insulin signaling cascade and more specifically on the activity of Akt kinase [36]. To establish the dependence of insulin degradation on IDE, we treated undifferentiated 3T3-L1 cells with an IDE inhibitor, bacitracin, and measure insulin degradation rates [37]. We found reduced insulin degradation rates with increasing concentrations of bacitracin (**Fig. 7E**). We further evaluated the impact of inhibiting insulin degradation on fat cell differentiation by adding bacitracin to differentiating 3T3-L1 cell cultures. We observed drastic inhibitions of lipid droplet formation with bacitracin treatments (**Fig. 7F**). However, bacitracin addition, even at high concentrations, did not completely inhibit insulin degradation or lipid droplet formation (**Fig. 7E, F**). This observation is consistent with previous reports where multiple insulin processing pathways are found [37]. Furthermore, lipid droplet formation is dependent on more factors than insulin degradation [3]. Indeed, Temple *et al.* reported that lipid droplet formation can also be suppressed in the presence of pyruvate, which serves as the extracellular energy source, despite the expression of adipogenic genes [38]. Insulin-stimulated phosphorylation of Akt was unaffected, which suggests that Akt-mediated downstream events including expression of IDE were unaffected. However, addition of glucose restored lipid droplet formation activity. Together, Temple *et al.* provided complementary evidence to our observation that adipogenic gene expression is insufficient to drive lipid droplet formation. Temple *et al.* showed that extracellular energy source can also control lipid droplet formation activity. Additionally, our data clearly indicate the importance of insulin degradation in lipid droplet formation. Rapid insulin degradation could enable recycling of surface insulin receptors leading to increased sensitivity to new source of insulin stimulation in lipid-rich cells (**Fig. 4D–F**) [32].

### Prolonged insulin stimulation leads to complete differentiation of cell cultures

**Figure 8:**
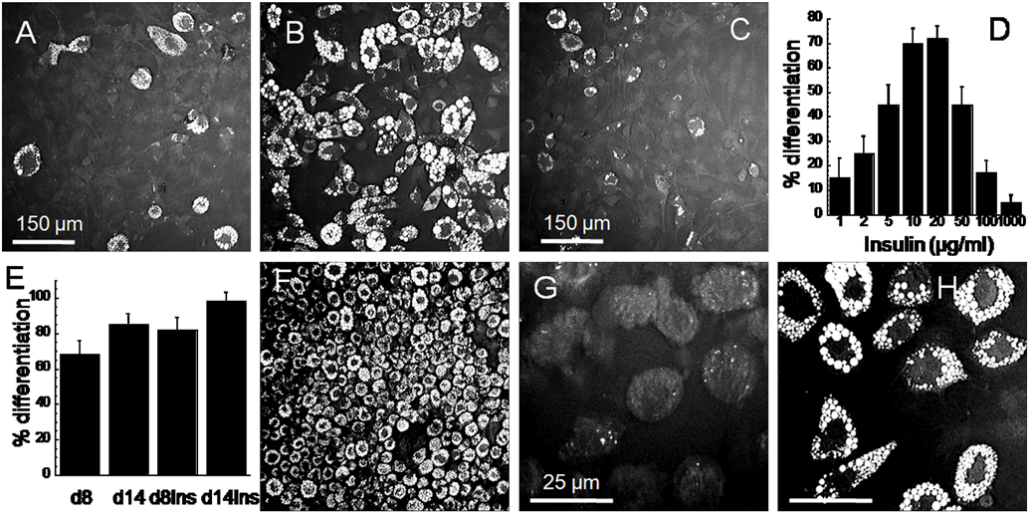
Lipid droplet accumulation as a function of insulin stimulation. Percentage differentiation of cells stimulated with (A) 1 µg/ml, (B) 10 µg/ml, and (C) 100 µg/ml insulin. Percentage differentiation is defined as the fraction of cells with cytoplasmic lipid droplet in a differentiating population. (D) Percentage differentiation as a function of insulin concentration. Error bars represent distribution across 4 repeated assays. (A–D) Cells are first treated with a constant standard concentration of IBMX/Dex mixture, then with varying insulin concentration and evaluate for percentage differentiation on day 8. (E) Percentage differentiation evaluated on day 8 and day 14 for cell populations stimulated with 10 µg/ml insulin from day 2 to day 4 (left 2 columns), and for cell population stimulated with 10 µg/ml insulin continuously (right 2 columns). (F) CARS image of a cell population stimulated with 10 µg/ml insulin continuously at day 14. (G) CARS image of a sorted lipid-poor cell population on day 8. (H) Lipid accumulation is observed on day 14, 6 days after sorting, in lipid-poor cell population with continuous 10 µg/ml insulin stimulation. Scale bars of 150 µm for panels A, B, C, F and 25 µm for panels G and H.

We further proved the significance of insulin signaling by examining the effects of insulin stimulation on cytoplasmic lipid droplet formation. First, by keeping IBMX and dexamethasone input stimulations constant from day 0 to day 2, we varied the concentrations of insulin in cell cultures from day 2 to day 4. Quantitative analysis revealed a direct correlation of lipid droplet formation on insulin stimulation at low insulin concentration from 1 µg/ml to 20 µg/ml (**Fig. 8A, B, D**). Inhibition of lipid droplet formation was observed at 50 µg/ml or higher insulin concentrations (**Fig. 8C, D**). Second, we prolonged insulin stimulation by replenishing differentiating cell cultures with new source of insulin at two-day intervals. We observed 100% of cells with lipid droplet accumulation could be accomplished with prolonged insulin stimulation at 14 days after adipogenesis induction (**Fig. 8E, F**). To show that continued insulin stimulation, by itself, is sufficient for lipid droplet accumulation, we added 10 µg/ml insulin to sorted lipid-poor cell populations at day 8 (**Fig. 8G**). We observed that all lipid-poor cells eventually accumulate lipid droplets on day 14 (**Fig. 8H**). Taken together, our data clearly show the dependence of cytoplasmic lipid droplet accumulation on insulin stimulation. By varying insulin concentration and duration of stimulation, the number of lipid-rich cells within differentiating populations can be reliably produced.

## Discussion

It has been proposed that the degree of complexity among organisms is not correlated with the number of encoded genes, but with the regulation of gene expression and protein network interaction [39]. In fact, many important processes including cell-fate determination and embryonic development are regulated by spatial-temporal interactions of DNA, RNA, protein, and other small molecules [40]. Fluctuations in the concentration of regulatory molecules can be used to select differentiation pathways [7], [41]. Moreover, cell-to-cell variability can be used to improve evolvability of a population [7], [42]. To investigate non-genetic contributions to cell behavior, single-cell measurement techniques are required to study in real time the dynamics of gene and protein network interaction [5], [9].

Here, we combine the multimodality imaging capability of a CARS microscope with the versatility of fluorescent proteins [43] and fluorescently labeled molecules to examine simultaneously multiple cellular events within a single cell. Using phenotypic transformation of 3T3-L1 cells into fat cells as a model system, adipogenic gene expression, insulin signaling, and glucose import are visualized simultaneously with lipid droplet accumulation. Flow cytometry further provides quantitative single-cell analysis in a high-throughput manner. We find that phenotypic variability among differentiating 3T3-L1 cells is dependent on the kinetics of an insulin signaling cascade. Expressions of early-stage and late-stage adipogenic marker genes strongly suggest that all cells in a differentiating 3T3-L1 cell culture are committed to fat cell differentiation (**Fig. 2B** **&** **Fig. 3**). However, differentiating cells exhibit tremendous cell-to-cell variability on insulin sensitivity, kinase activity, glucose import, and expression of an insulin degradation enzyme (**Fig. 4** **–** [Fig pone-0005189-g005] [Fig pone-0005189-g006] **Fig. 7** **,** **Fig. S6**). It is important to point out that these variables are part of an insulin signaling cascade (**Fig. 1A**) [44]. Increased insulin sensitivity in lipid-rich cells is associated with increased kinase activity, glucose import, and insulin degradation. The combinatorial effect could conceivably amplify subtle variability in insulin signal processing into drastic variation in the degree of cytoplasmic lipid droplet accumulation. Indeed, increase concentration and duration of insulin stimulation are positively correlated with the number of cells with cytoplasmic lipid droplet accumulation (**Fig. 8**). The observed dependence of lipid droplet accumulation on the kinetics of an insulin signaling cascade is also consistent with many previous studies where heterogeneous kinase activities are reported in differentiating 3T3-L1 cells [45] and where increased insulin-like growth factor overcome Pref-1 mediated inhibition of adipocyte differentiation [46]. Many recent studies on prokaryotic cells identify stochastic expression or activity of a single protein as the cause of phenotypic and behavioural variability [5], [7], [9], [10]. It is conceivable that stochasticity of an insulin signaling cascade could contribute to the variability of lipid droplet formation during adipogenesis. Future studies of insulin signaling pathway could potentially identify specific events or molecules which control the rate of lipid droplet formation. Such insights should advance our understanding of the molecular control of adipogenesis and enable better drug design to interfere with adipogenesis.

As endocrine organs whose secreted adipokines influence whole body physiology and metabolism, adipose tissues have become a major drug target for obesity treatment [2], [3]. However, heterogeneity in adipogenesis interferes with the efficacy of drugs aiming at adipocyte differentiation. Understanding the source of cell-to-cell variability during adipogenesis should improve drug design and evaluation strategy. Single-cell reporter systems described in this paper and elsewhere [47] should provide a rapid means to directly evaluate specific effects of drug on adipogenic gene expression and insulin signaling pathway. Furthermore, high penetration depth and three-dimensional sectioning capability intrinsic to CARS microscopy have allowed its application to tissue and live animal imaging [13]. Thus, single-cell studies of heterogeneity in drug-inducible adipogenesis in 3T3-L1 cells in cultures could be readily translated to single-cell studies of heterogeneity in adipogenesis of implanted cells in live animals [48], [49]. Finally, signaling cascade is a common gateway for intracellular signaling pathways [50], [51]. Variability in the cascade of responses could divide a genetically identical cell population into phenotypically heterogeneous populations with varying rates of growth, proliferation, survival, and motility [51]. It is conceivable that the single-cell molecular profiling approach described in this paper can be applied to study variability of signaling cascades in diseases to uncover dynamic properties inaccessible to population measurements.

## Materials and Methods

### A multimodal CARS microscope

A multimodal microscope which allows CARS and TPEF imaging on the same platform has been previously described [23]. For forward CARS imaging of lipids, the wave number difference between pump laser and Stokes laser is tuned to 2840 cm^−1^ which matched the Raman shift of symmetric CH_2_ stretch vibration. For TPEF imaging of fluorescent molecules, back reflected signal was collected by the same objective, spectrally separated from the excitation source, transmitted through appropriate bandpass filters, and detected by a photomultiplier tube (PMT, H7422-40, Hamamatsu, Japan) mounted at the back port of the microscope. Bandpass filters 520/40 nm and 600/65 nm (Chroma Technologies, Rockingham, VT) were used to transmit TPEF signals from GFP and RFP or Cy3, respectively. To remove epi-reflected CARS signals from TPEF signals of Cy3 and RFP, the timing of the pump and Stokes lasers are desynchronized. Acquisition time for each image was 1.12 s. Images were analyzed using FluoView software (Olympus, Center Valley, PA).

### Inducing adipogenesis in 3T3-L1 cells

Adipogenesis was induced using an adipogenesis assay kit (Cat. No. ECM 950, Chemicon International, Temecula, CA). 3T3-L1 cells were grown to confluence in DMEM media consisting of 25 mM of glucose supplemented with 10% calf serum and penicillin/streptomycin. On day 0, cells were induced with initiation media (0.5 mM IBMX and 1 µM dexamethasone in DMEM media supplemented with 10% fetal calf serum and penicillin (100 units/ml)/streptomycin (100 µg/ml). On day 2, initiation media was replaced with progression media (10 µg/ml insulin in DMEM media supplemented with 10% fetal calf serum and penicillin/streptomycin). On day 4, progression media was replaced with maintenance media (DMEM supplemented with 10% fetal calf serum and penicillin/streptomycin). From day 4 to day 8, cells were kept in maintenance media with new maintenance media being replaced by every two days. Cells are incubated at 37°C with 5% CO_2_ at all time.

### Real-time PCR adipogenic gene expression profiling

Total RNA was extracted using RNAqueous kit (Cat. No. AM1912, Ambion, Austin, Texas). A RT^2^ SYBR Green qPCR Master Mix (Cat. No. PA-011, SABiosciences, Frederick, MD) was employed. Real-time PCR was performed using the iQ5 real-time PCR detection system (BioRad, Hercules, CA) according to SABiosciences suggested protocol. Forward and reverse primers for PPARγ, Pref-1, aP2, C/EBPα, LP2, and a house-keeping gene acidic ribosomal phosphoprotein OP were purchased from Integrated DNA Technologies (Coralville, IA) using previously published sequences [52].

### A dual-reporter plasmid to study Pref-1 and PPARγ gene expression

A ∼2 kb DNA fragment was generated by total synthesis (Genscript, Piscataway, NJ) and cloned into a pDSRed-Express-DR plasmid (Cat. No. 632423, Clontech, Mountain View, CA) between restriction sites XhoI and BamHI. This DNA fragment comprises: a 0.6 kb fragment of PPARγ promoter (nucleotides −603 to +62) controlling the expression of a destabilized variant of green fluorescent protein (0.8 kb), a SV40 polyA transcription termination signal (0.25 kb), and a 0.2 kb fragment of Pref-1 promoter (nucleotides −183 to +25). The excitation and emission maxima for green fluorescent protein are 496 nm and 506 nm, and for red fluorescent protein are 557 nm and 579 nm, respectively.

### Stable transfection of 3T3-L1 cells

3T3-L1 cells at density of 1×10^5^ in 35 mm culture dishes were transfected with plasmid DNA using Fugene®6 transfection reagent (Cat. No. 11815091001, Roche Diagnostics, Indianapolis, IN) at the ratio of 6 µl of Fugene®6 reagent to 1 µg DNA. At 24 hours after transfection, cells were selected with 400 ng/ml Geneticin® G418 Sulfate (Cat. No. 10131, Invitrogen, Carlsbad, CA) until individual colonies were observed under a microscope. Colonies were detached from culture dishes by incubation with non-enzymatic cell dissociation reagent (Cat. No. 1676949, MP Biomedicals, Solon, OH) for 5 minutes at 37°C. Individual colonies were carefully removed using pipette tips under a microscope. To ensure a single clone is used, a selected colony was diluted into multi-well plates at the concentration of one cell per well and re-grown in 100 ng/ml Geneticin® G418 Sulfate (Cat. No. 10131, Invitrogen, Carlsbad, CA).

### Insulin imaging assay

Insulin-Cy3 (Cat. No. FC303506, Phoenix Pharmaceuticals, Burlingame, CA) was used for all imaging experiments.

### PI3K activity assay

PI3K assay was performed using a PI3K assay kit according to manufacturer's protocol (Cat. No. EK 2010, Panomics, Redwood City, CA).

### Fluorescent glucose analog import assay

0.3 mM of fluorescent glucose analog (Cat. No. N23106, Invitrogen, Carlsbad, CA) in glucose-free media was added to differentiating cell cultures for 1 minute. Cell cultures were thoroughly washed to remove all exogenous fluorescent glucose analog, then imaged for intracellular fluorescent signal or analyzed with flow cytometry.

### Flow cytometry sorting and analysis. *Cell sorting*

An EPICS ALTRA flow cytometer was used to sort differentiating 3T3-L1 cells (Beckman-Coulter, Fullerton, CA). Cells were dissociated from culture vessels with pre-warmed cell dissociation solution, passed through a 60 µm filter, and subjected to flow cytometry sorting. Lipid-poor cells were defined as those with side scattering signals below 200. Lipid-rich cells were those with side scattering signals above 200. Sorted cells were collected in supplemented DMEM media and subjected to real-time PCR gene expression profiling or kinase activity assay. Insulin-FITC (Cat. No. I2383, Sigma Aldrich, St Louis, MO) was used instead of insulin-Cy3 due to the availability of the 488 nm excitation laser source of the cytometer. No difference in cell response to labelled insulin sources was observed.

### IDE immuno-labeling

Rabbit polyclonal primary antibodies to IDE (Cat. No. AB25970, Abcam, Cambridge, MA) and goat polyclonal to rabbit IgG conjugated to FITC secondary antibodies (Cat. No. AB6717, Abcam, Cambridge, MA) were used according to manufacturer's protocols.

## Supporting Information

Figure S1 3T3-L1 cell percentage differentiation as a function of cell source and cell passage number. 3T3-L1 cell lines are obtained from American Type Culture Collection (ATCC) and from 3 different research labs. Cells at passage 2 are used to evaluate percentage differentiation among difference cell sources. ATCC cells are also evaluated at different cell passage number. In all experiments, post-confluence cells are first treated with a mixture of 0.5 mM IBMX and 1 µM dexamethasone from day 0 to day 2. Then 10 µg/ml of insulin are added from day 2 to day 4. Percentage differentiation is evaluated on day 8 after adipogenesis induction. Percentage differentiation is calculated based on the number of lipid-rich cells in a differentiating population identified with CARS microscopy.
(0.23 MB TIF)

Figure S2 Flow cytometry sorted cell populations. An EPICS ALTRA flow cytometer was used to sort differentiating 3T3-L1 cells (Beckman-Coulter, Fullerton, CA). Cells are dissociated from culture vessels with pre-warmed cell dissociation solution, passed through a 60 µm filter, and subjected to flow cytometry sorting. Lipid-poor cells are defined as those with side scattering signals below 200. Lipid-rich cells are those with side scattering signals above 200. Sorted cells are collected in supplemented DMEM media. Images acquired with CARS microscopy.
(0.42 MB TIF)

Figure S3 Evaluating the effect of a PPARγ agonist, rosiglitazone, on cell differentiation. (A–D) CARS image of cell cultures treated with varying concentrations of rosiglitazone. (E) Quantitative analysis of percentage differentiation as a function of rosiglitazone concentration. Error bars represent distribution across 4 repeated experiments. Rosiglitazone addition appears to have no impact on the percentage of 3T3-L1 cells with cytoplasmic lipid droplet accumulation.
(1.77 MB TIF)

Figure S4 Competitive cellular uptake of labeled insulin and glucose analog. (A) Competitive binding of labeled insulin versus unlabeled insulin. Undifferentiated cells at day 0 are first treated with varying concentration of unlabeled insulin for 5 minutes, then treated with labeled insulin at 10 µg/ml for 5 minutes and subjected to flow cytometry analysis. (B) Competitive cellular uptake of fluorescent glucose analog versus glucose. Undifferentiated cells at day 0 are first treated with varying concentration of glucose for 5 minutes, then treated with fluorescent glucose analog at 0.3 mM for 1 minute and subjected to flow cytometry analysis. Average fluorescence intensities of 2000 cells at each concentration are presented. Error bars represent distribution across 4 repeated experiments.
(0.20 MB TIF)

Figure S5 Insulin binding and glucose analog uptake as functions of days after induction. (A) Labeled insulin binding as a function of days after induction. 3T3-L1 cells at specified days are treated with 10 ug/ml of labeled insulin for 5 minutes and subjected to flow cytometry analysis. (B) Fluorescent glucose analog uptake as a function of days after induction. 3T3-L1 cells at specified days are first washed with glucose-free media for 2 hours, then treated with 0.3 mM of fluorescent glucose analog for 1 minute and subjected to flow cytometry analysis. Average fluorescence intensities of 2000 cells at each specified day are presented. Error bars represent distribution across 4 repeated experiments. On the x-axis, 4lp, 6lp, and 8lp represent lipid-poor cell populations sorted with flow cytometry on day 4, day 6, and day 8, respectively. On the x-axis, 4lr, 6lr and 8lr represent lipid-rich cell populations sorted with flow cytometry on day 4, day 6, and day 8, respectively.
(0.24 MB TIF)

Figure S6 Lipid droplets formation as a function of PPARγ gene activity, insulin uptake, and glucose import. Y-axis reports single-cell flow cytometry analysis of lipid droplets formation, or side scatterings due to cytoplasmic granularity. X-axis reports fluorescence intensity of dsGFP, insulin-Cy3, and glucose analog-FITC which reports PPARγ gene activity, insulin uptake, and glucose import, respectively. Cells are analyzed at day 8 after adipogenesis induction. Lipid-rich cells exhibit a lack of correlation between PPARγ gene activity and lipid droplet formation, but strong correlations between lipid droplet formation, insulin uptake, and glucose import.
(0.74 MB TIF)
