# Supplementary figures and images for: Single-Cell Profiling Reveals the Origin of Phenotypic Variability in Adipogenesis
Source: PLoS One. 2009 Apr 9;4(4):e5189. doi: 10.1371/journal.pone.0005189 (PMC2663816; doi:10.1371/journal.pone.0005189)

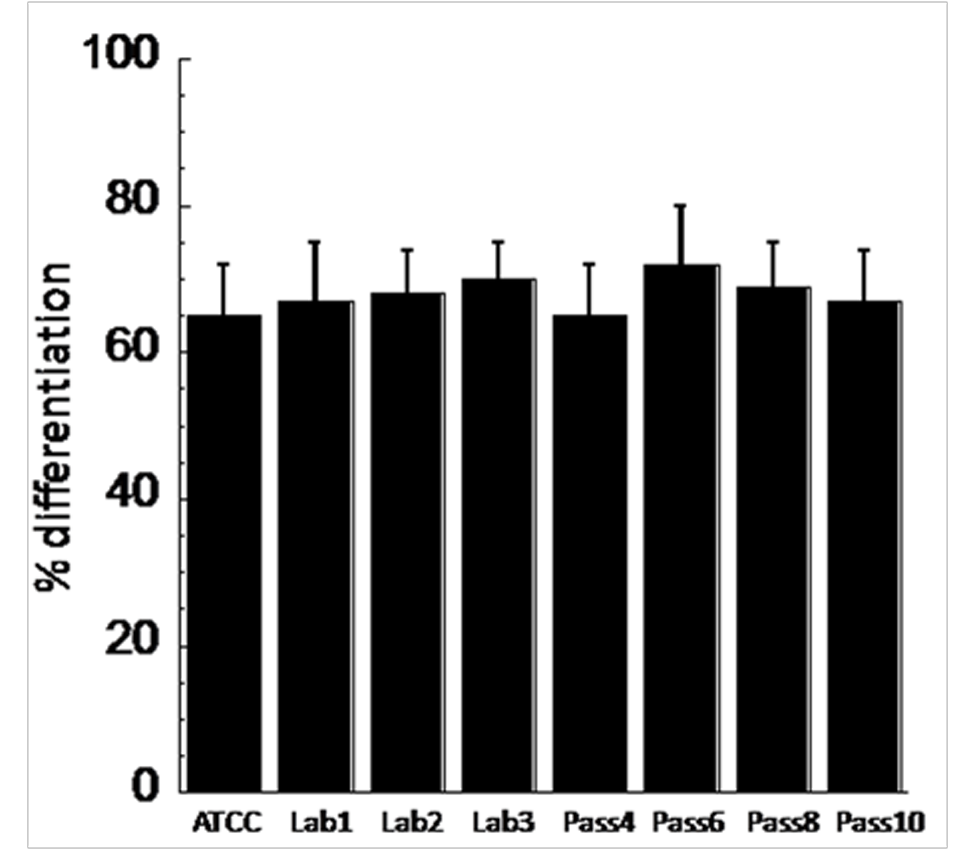

Supplement: Figure S1 — 3T3-L1 cell percentage differentiation as a function of cell source and cell passage number. 3T3-L1 cell lines are obtained from American Type Culture Collection (ATCC) and from 3 different research labs. Cells at passage 2 are used to evaluate percentage differentiation among difference cell sources. ATCC cells are also evaluated at different cell passage number. In all experiments, post-confluence cells are first treated with a mixture of 0.5 mM IBMX and 1 µM dexamethasone from day 0 to day 2. Then 10 µg/ml of insulin are added from day 2 to day 4. Percentage differentiation is evaluated on day 8 after adipogenesis induction. Percentage differentiation is calculated based on the number of lipid-rich cells in a differentiating population identified with CARS microscopy. (0.23 MB TIF) [file pone.0005189.s001.tif]

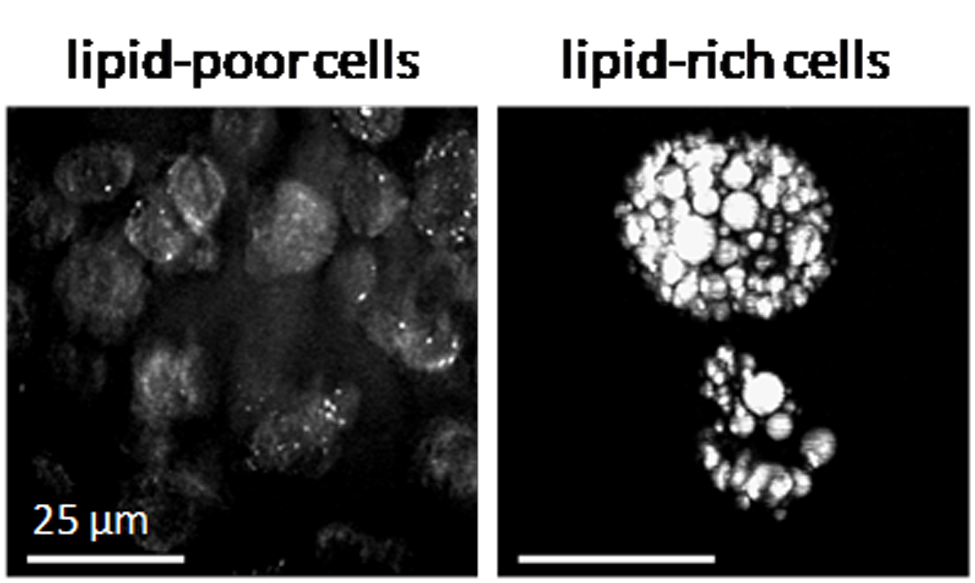

Supplement: Figure S2 — Flow cytometry sorted cell populations. An EPICS ALTRA flow cytometer was used to sort differentiating 3T3-L1 cells (Beckman-Coulter, Fullerton, CA). Cells are dissociated from culture vessels with pre-warmed cell dissociation solution, passed through a 60 µm filter, and subjected to flow cytometry sorting. Lipid-poor cells are defined as those with side scattering signals below 200. Lipid-rich cells are those with side scattering signals above 200. Sorted cells are collected in supplemented DMEM media. Images acquired with CARS microscopy. (0.42 MB TIF) [file pone.0005189.s002.tif]

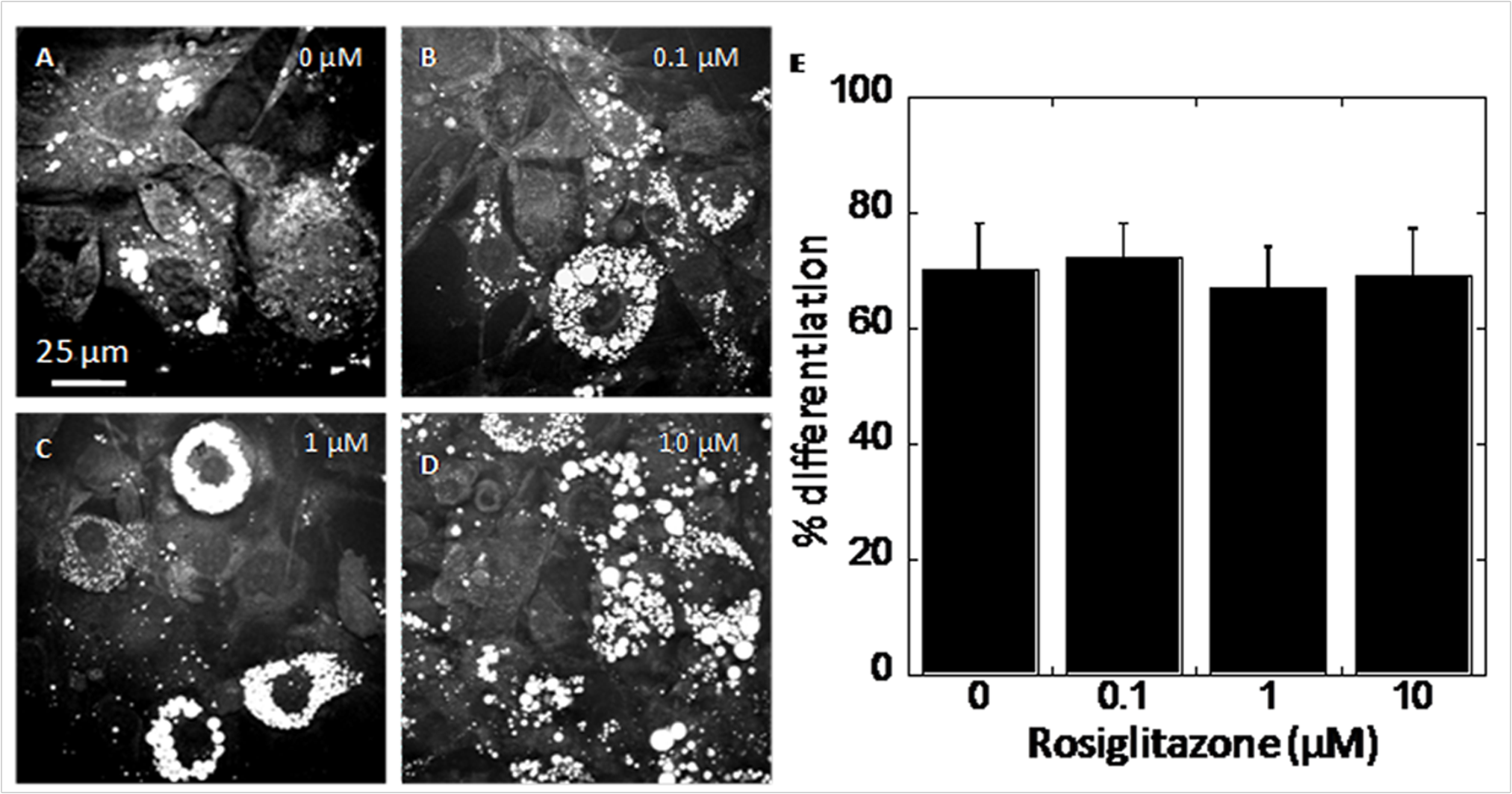

Supplement: Figure S3 — Evaluating the effect of a PPARγ agonist, rosiglitazone, on cell differentiation. (A–D) CARS image of cell cultures treated with varying concentrations of rosiglitazone. (E) Quantitative analysis of percentage differentiation as a function of rosiglitazone concentration. Error bars represent distribution across 4 repeated experiments. Rosiglitazone addition appears to have no impact on the percentage of 3T3-L1 cells with cytoplasmic lipid droplet accumulation. (1.77 MB TIF) [file pone.0005189.s003.tif]

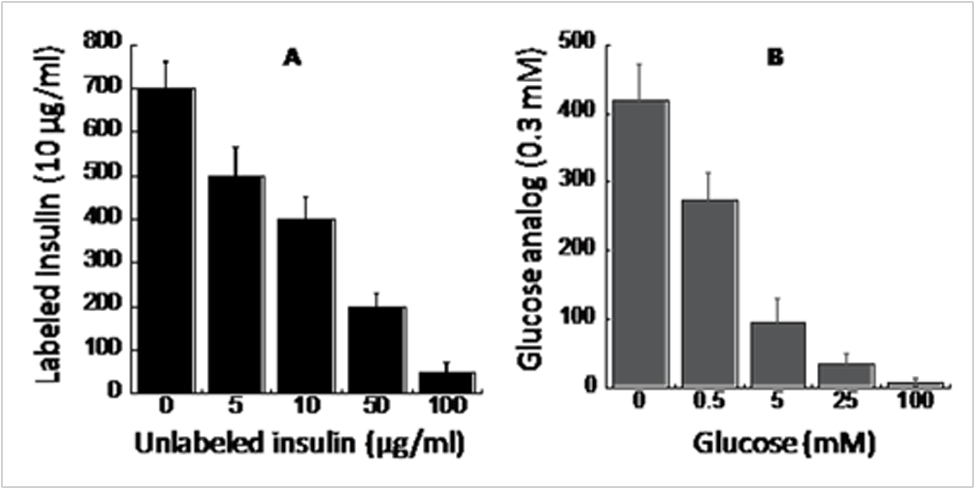

Supplement: Figure S4 — Competitive cellular uptake of labeled insulin and glucose analog. (A) Competitive binding of labeled insulin versus unlabeled insulin. Undifferentiated cells at day 0 are first treated with varying concentration of unlabeled insulin for 5 minutes, then treated with labeled insulin at 10 µg/ml for 5 minutes and subjected to flow cytometry analysis. (B) Competitive cellular uptake of fluorescent glucose analog versus glucose. Undifferentiated cells at day 0 are first treated with varying concentration of glucose for 5 minutes, then treated with fluorescent glucose analog at 0.3 mM for 1 minute and subjected to flow cytometry analysis. Average fluorescence intensities of 2000 cells at each concentration are presented. Error bars represent distribution across 4 repeated experiments. (0.20 MB TIF) [file pone.0005189.s004.tif]

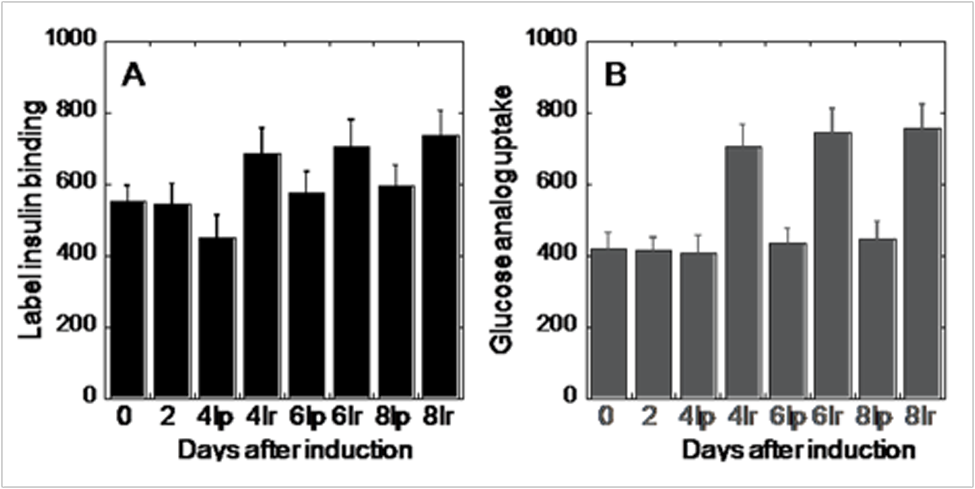

Supplement: Figure S5 — Insulin binding and glucose analog uptake as functions of days after induction. (A) Labeled insulin binding as a function of days after induction. 3T3-L1 cells at specified days are treated with 10 ug/ml of labeled insulin for 5 minutes and subjected to flow cytometry analysis. (B) Fluorescent glucose analog uptake as a function of days after induction. 3T3-L1 cells at specified days are first washed with glucose-free media for 2 hours, then treated with 0.3 mM of fluorescent glucose analog for 1 minute and subjected to flow cytometry analysis. Average fluorescence intensities of 2000 cells at each specified day are presented. Error bars represent distribution across 4 repeated experiments. On the x-axis, 4lp, 6lp, and 8lp represent lipid-poor cell populations sorted with flow cytometry on day 4, day 6, and day 8, respectively. On the x-axis, 4lr, 6lr and 8lr represent lipid-rich cell populations sorted with flow cytometry on day 4, day 6, and day 8, respectively. (0.24 MB TIF) [file pone.0005189.s005.tif]

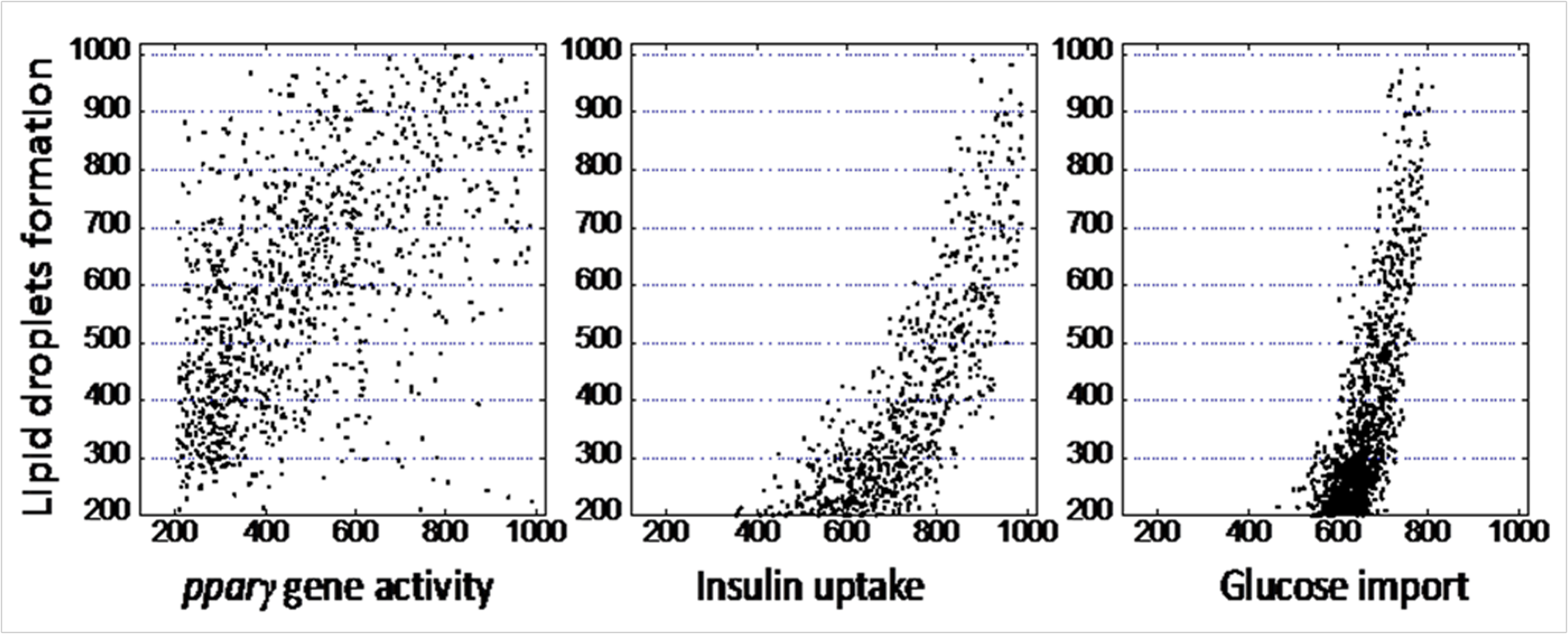

Supplement: Figure S6 — Lipid droplets formation as a function of PPARγ gene activity, insulin uptake, and glucose import. Y-axis reports single-cell flow cytometry analysis of lipid droplets formation, or side scatterings due to cytoplasmic granularity. X-axis reports fluorescence intensity of dsGFP, insulin-Cy3, and glucose analog-FITC which reports PPARγ gene activity, insulin uptake, and glucose import, respectively. Cells are analyzed at day 8 after adipogenesis induction. Lipid-rich cells exhibit a lack of correlation between PPARγ gene activity and lipid droplet formation, but strong correlations between lipid droplet formation, insulin uptake, and glucose import. (0.74 MB TIF) [file pone.0005189.s006.tif]
